# Stability of Weld Pool and Elimination of Weld Defects in Aluminum Alloy Plasma Arc Keyhole Welding at Continuously Varying Positions

**DOI:** 10.3390/ma14195898

**Published:** 2021-10-08

**Authors:** Wei Cheng, Xinqiang Ma, Junlin Zhang, Zhaoyang Yan, Fan Jiang, Shujun Chen

**Affiliations:** 1 Faculty of Materials and Manufacturing, Beijing University of Technology, Beijing 100124, China; chengweijob@163.com (W.C.); maxin-qiang@163.com (X.M.); jiangfan@bjut.edu.cn (F.J.); shujchen@bjut.edu.cn (S.C.); 2 Laser Institute, Qilu University of Technology (Shandong Academy of Sciences), Jinan 250353, China; 3 Department of Spacecraft Environmental Engineering, China Academy of Space Technology, Beijing 100124, China; xingzheng_welder@163.com; 4 Engineering Research Centre of Advanced Manufacturing Technology for Automotive Components, Ministry of Education, Beijing University of Technology, Beijing 100124, China

**Keywords:** VPPA, weld defects, keyhole welding, aluminum alloy, tensile strength

## Abstract

Mathematical statistics were used to study the stability of weld pool and the elimination of weld defects in aluminum alloy plasma arc keyhole welding at continuously varying positions. In the mathematical model, the mass transfer position and spatial welding position were taken as the input, and the shape of the welded joints (symmetry/deviation) was taken as the output. The results showed that the fitted curves of the front, back, and average deviations of the weld seam were all similar to the actual curves. According to the optimum results obtained in the experiment and the mathematical models, the mass transfer position only needs to be adjusted once (near to 30°) during the continuously varying positions, from vertical-up to horizontal welding. A breakthrough from fixed environmental variables to dynamic environmental variables in the process control of the keyhole weld pool was realized, which enabled the Al-alloy keyhole weld pool to resist the disturbance caused by gravity during variable position welding. The deviation of the welded joints of the whole plate was smaller than 0.5 mm, and the mechanical properties of the weld reached at least 85% compared to those of the base material, thus meeting the requirements of Al-alloy welding.

## 1. Introduction

As a high-performance metal for lightweight equipment, aluminum alloys are widely used in manufacturing applications in the aerospace and automotive industries due to their high specific strength and good thermal stability [1,2]. The development of Al-alloy manufacturing industries is inseparable from the processing technologies used, especially the joining process of welding [3]. Combining the advantages of high energy beams and variable polarity gas tungsten arcs, variable polarity plasma arc (VPPA) welding is a “zero-defect” process for Al-alloy welding [4,5,6]. However, application of VPPA is limited in vertical-up welding. Based on the specific problems of high-precision spacecraft manufacturing and the increasing size of welded structures, the development of in situ welding technologies to ensure prioritization of the posture of workpieces is urgent.

VPPA vertical-up welding has several advantages: (1) the plasma jet creates a keyhole through the entire work-piece that increases the gas-liquid interface of the weld pool, thereby greatly increasing the possibility of gas escaping; (2) the keyhole weld pool avoids the high temperature zone of the heat source when the plasma jet pushes away the metal, and the gas-liquid interface of the weld pool remains within a stable temperature zone, which is favorable for ensuring that the liquid at the rear side of the weld pool integrates steadily and forms a weld seam [7,8,9,10]. In nonvertical-up welding, the lack of gravity-assisted molten pool flow can cause asymmetric metal flows [11,12,13,14], resulting in asymmetric welds and a decreased weld quality.

Studies have shown that by reasonably adjusting and matching the thermal output of the VPPA, the requirements of welding assemblies in multiple fixed positions (e.g., horizontal welding, vertical-up welding, and flat welding) can be realized, which greatly broadens the application scope of this type of welding [11,12,13,15,16]. However, when the position is continuously changed during welding, gravity dynamically influences the keyhole weld pool and makes it difficult to maintain the quasi-stable state of fixed position welding, which becomes unstable during welding, and the metal melt behind the weld pool fluctuates greatly, which can cause intermittent closure or even a lack of closure, thus resulting in uneven weld morphologies and continuous cutting [17]. In addition, in the process of welding, continuously changing the spatial position of the keyhole weld pool negatively impacts the escape of gas, leading to defects in enrichment that severely affect the strength of the entire structure.

**Figure 1:**
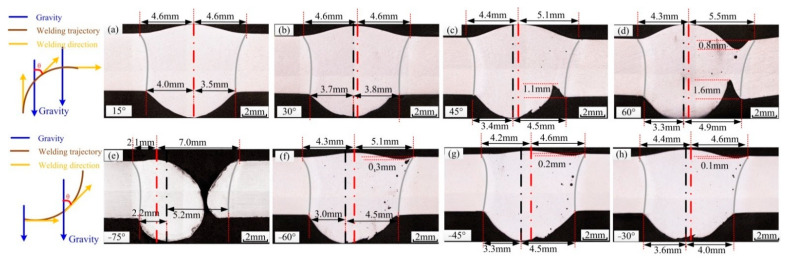
Cross section of the welded joints before optimization, red line is the center line of weld surface, black line is the center line of weld root-surface. (**a**) 15°, (**b**) 30°, (**c**) 45°, (**d**) 60°, (**e**) −75°, (**f**) −60°, (**g**) −45°, (**h**) −30°.

Figure 1 shows the welded joints when the welding position is continuously varied to different angles, where the angle shown is that between the direction of gravity and the welding direction. Figure 1a–d shows the cross-section from vertical-up welding to horizontal welding, where the angle between two welded joints is approximately 15°. As shown in Figure 1a, in vertical-up welding, the welding joint is symmetric, with no deviation in the welding seam. As the angle increases, the deviation becomes increasingly obvious. When the angle is approximately 45°, an undercut of 1.1 mm appears on the back of the weld. When the angle is approximately 60°, undercutting appears on both sides of the weld. As the angle continues to increase, the thin liquid metal bridge on the weld pool cannot withstand gravity, and thus, the weld pool collapses, and the weld is cut. Following the cross-section from horizontal welding to vertical-up welding, the weld pool is not able to form the liquid bridge between −90° and −70°, and the weld seam does not easily form. At −70°, the liquid bridge is formed at the solidification side, but it is unstable. In the process of variable position welding, if the metal liquid bridge is formed, the entire process can be completed, but with a low quality.

Previous studies have investigated the stability of the keyhole weld pool, the flow behavior of liquid metal and the stagnation point, the mechanisms of the formation and elimination of porosity defects, and the optimization of welded joint asymmetry and have revealed the mechanism of defect formation in variable-position welding and weld-seam optimization [2,13,17]. However, welding in which the position is continuously varied has not been studied.

In this paper, the method of mathematical statistics was used. More specifically, the response surface methodology (RSM), based on a central composite design (CCD) and the Design-Expert software (V12.0) were used to design the experiment and establish mathematical models to verify the applicability of continuously varying the welding position [18,19,20,21]. Weld undercut and cutting, porosity, and asymmetric welds are the main problems in VPPA Al-alloy keyhole welding when the welding position is continuously varied. The formation of welds and porosity can be solved via parameter optimization, and asymmetric weld performance is currently the most challenging problem. Thus, an asymmetric welded joint was taken as the main research object in this study. In the mathematical model, the mass transfer position and spatial welding position were taken as the input, and the shape of the welded joints (symmetry/deviation) was taken as the output. Based on the mathematical model, the optimal symmetry of welded joints was determined. Finally, the process optimization strategy obtained in this study was implemented in the variable position welding of a 5A06 Al-alloy, and the mechanical properties of the welded joints were analyzed. The results provide a theoretical basis for eliminating the influence of a continuously varying gravitational force on the weld pool in variable position welding.

## 2. Materials and Methods

5A06 Al-alloy with good corrosion resistance, machinability, and weldability, is one of the mostly widely used materials for automotive, aerospace, and other industries [22,23]. In this paper, plates with a size of 750 mm × 400 mm × 5 mm were selected as the base material (BM), which was provided by Weihai Jindi Non-ferrous Metals Co., Ltd. (Weihai, China) [23], the chemical compositions are shown in Table 1. The wire used in this paper was ER5183 and the diameter was 1.2 mm, which has a similar chemical composition in comparison with the BM [22]; the chemical compositions are shown in Table 1.

### 2.1. Experimental Setup

**Figure 2:**
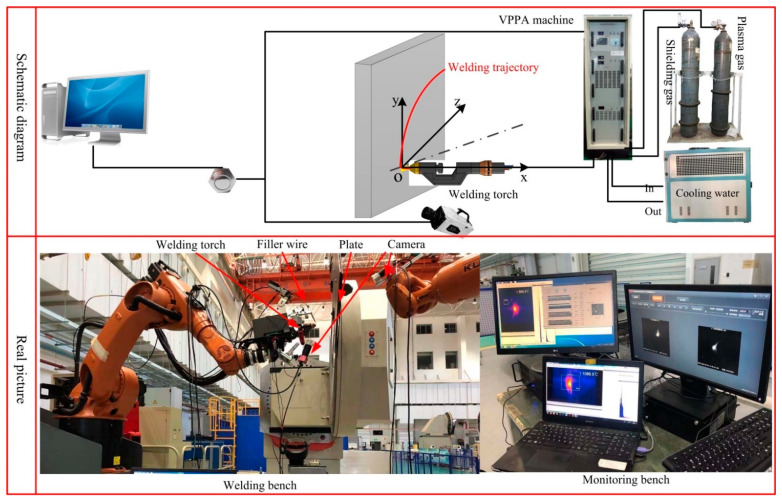
Observation system for liquid metal flow of VPPA horizontal welding.

To measure and study the stability of the weld pool and elimination of weld defects in aluminum alloy plasma arc keyhole welding at continuously varying positions, a test bed was established (shown in Figure 2). The welding power source in this system was made by Beijing University of Technology (VPPA-500, Beijing University of Technology, Beijing, China). The welding system included two argon gas cylinders for the shielding gas and plasma gas, a water-cooling tank, and a welding torch. The main welding parameters including welding current: DCEN (direct current electrode negative) and DCEP (direct current electrode positive), travel velocity, wire feed speed and gas flow rate are shown in Table 2. The selected parameters in Table 2 were based on a large number of tests, which were the optimal parameters to meet the stability of the keyhole molten pool and the quality of the weld formation. The keyhole molten pool will expand and became unstable when the welding current is increased, WFS decreased and travel speed decreased. Poor weld formation will appear due to the small heat input caused by decreasing welding current, increasing WFS, and increasing travel speed.

Tensile strength is one of the most used methods to evaluate mechanical properties. In this paper, the tensile tests were carried out using a universal testing machine INSTRON-5569 (Instron Limited, High Wycome, England) under standard NoGBT228–2002 of P.R. China [2,24], and the value used was the average value of three tests.

### 2.2. Definition of Welding Defects

**Figure 3:**
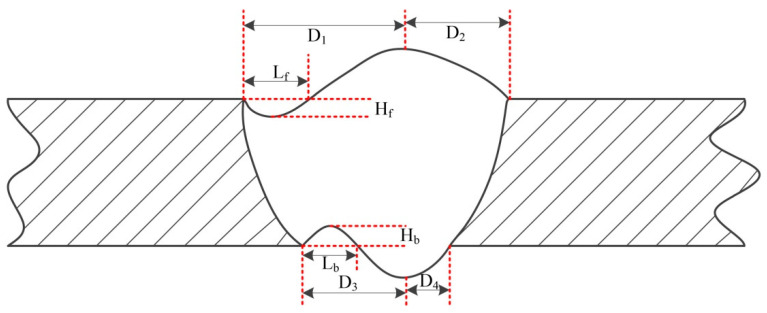
Schematic parameters typifying welding defects.

The difficulties of variable position plasma arc Al-alloy welding include the stable establishment of the keyhole weld pool, the weld pool flow, and the symmetry of the weld seam. This study applies an asymmetric heat source and asymmetric mass transfer to deal with the asymmetric pool flow caused by continuous variable gravity conditions without changing the welding parameters, that is, to reduce the impacts on the weld pool. To represent the influence of asymmetric weld pool flow on the weld formation, the following variables are defined (Figure 3): front deviation (D_f_), back deviation (D_b_), front undercut degree (U_f_) and back undercut degree (U_b_). Welding defects are shown by Equations (1)–(5).

(1) $$D_{f}=\left|D_{1}-D_{2}\right|$$

(2) $$D_{b}=\left|D_{3}-D_{4}\right|$$

(3) $$\bar{D}=\frac{D_{f}+D_{b}}{2}$$

(4) $$U_{f}=\int_{0}^{L_{f}}H_{f}dL\approx \frac{L_{f}+H_{f}}{2}$$

(5) $$U_{b}=\int_{0}^{L_{b}}H_{b}dL\approx \frac{L_{b}+H_{b}}{2}$$

where D_1_ and D_2_ are the distances from the maximum front excess weld metal height to the two weld boundaries; D_3_ and D_4_ are the distances from the maximum back excess weld metal height to the two weld boundaries; L_f_ and H_f_ are the length and depth of the front undercut, respectively; and L_b_ and H_b_ are the length and depth of the back undercut, respectively.

The greater the D_f_ and D_b_ values are, the more severe the weld asymmetry; the larger the U_f_ and U_b_ values are, the more severe the undercut.

## 3. Mathematical Model of Variable Position Welding

### 3.1. Establishment of a Welding Parameter Matrix

The aim of this study is to achieve variable position welding and improve the weld quality. The main factors affecting the quality of welded joints are the mass transfer position and the spatial welding position. Based on the schematic diagram of the central composite rotatable design (CCRD) parameter matrix, the upper and lower limits, the upper and lower levels and the centre point of each factor were normalized and coded according to five levels: −1.414, −1, 0, +1 and +1.414. The coded values should meet the following requirement, as shown in Equation (6):

(6) $$Z_{i}=\frac{\left(Z_{\max}-Z_{\min}\right)\left(X-X_{\min}\right)}{X_{\max}-X_{\min}}+Z_{\min}$$

where Z_i_ is the value of the normalized variable, Z_max_ is the maximum of the normalized variable, Z_min_ is the minimum of the normalized variable, X is the level of the factor, X_min_ is the upper limit of the factor level X, X_max_ is the lower limit of the factor level X.

According to the experiment, the welding parameters were determined, as shown in Table 2. Table 3 shows the relationship between the coded values of the factor levels and the actual values. The output of the regression model was the asymmetry of the welded joints, i.e., the deviation of the front excess weld metal height, the deviation of the back excess weld metal height, and the average deviation.

**Figure 4:**
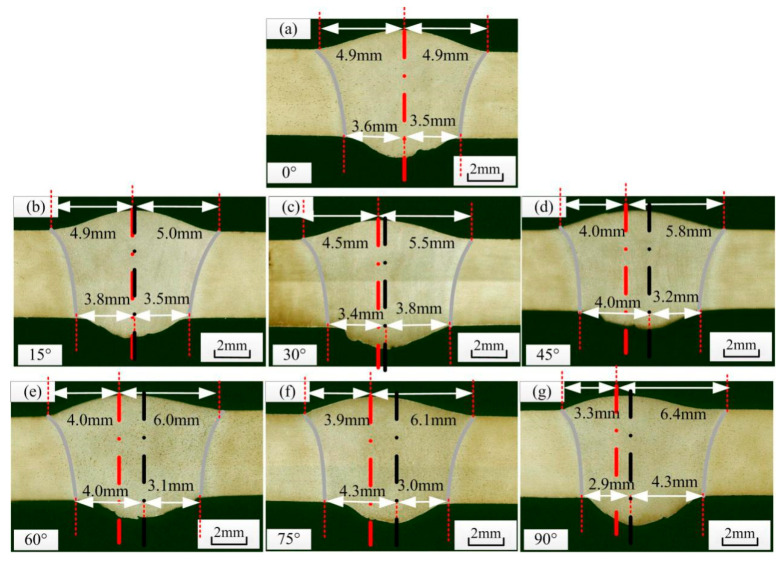
Welding defects with central mass transfer, red line is the center line of weld surface, black line is the center line of weld root-surface. (**a**) 0°, (**b**) 15°, (**c**) 30°, (**d**) 45°, (**e**) 60°, (**f**) 75°, (**g**) 90°.

**Figure 5:**
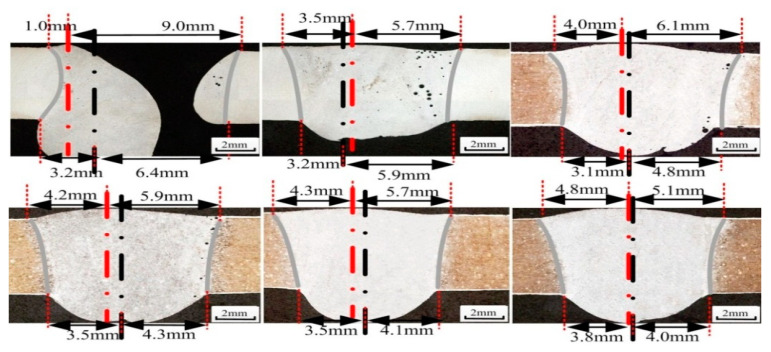
Welding defects with different mass transfer positions, red line is the center line of weld surface, black line is the center line of weld root-surface.

The asymmetry of welded joints seriously affects the weld quality. Figure 3 shows the definition of asymmetry for welded joints. According to the parameters in Table 3, weld seams with different mass transfer and welding positions were measured, and the front deviation, back deviation and average deviation of the welded joints were recorded (Table 4). Figure 4 shows the welding defects with central mass transfer, and Figure 5 shows the welding defects of welded joints with different mass transfer positions.

### 3.2. Establishment and Verification of the Welding Model

The test data in Table 4 were analyzed using regression analysis, and the relationship between the input and the output was established. The output included the front deviation, the back deviation, and the average deviation of the welded joints. Three mathematical models were established for the input and each output. In this study, the high-order regression equation between the spatial welding position and the mass transfer position, as well as the deviation of the weld seam were used:

This section may be divided by subheadings. It should provide a concise and precise description of the experimental results, their interpretation, as well as the experimental conclusions that can be drawn, as shown in Equations (7)–(9).

(7) $$\sqrt{y_{1}}=0.96+0.74T-0.20M-1.46T^{2}-1.66M^{2}+0.17T^{3}-1.91M^{3}+1.01T^{4}+0.94M^{4}$$

(8) $$\sqrt{y_{2}}=0.36+0.58T-0.89M+0.15T^{2}-0.40M^{2}$$

(9) $$\sqrt{y_{3}}=0.78+0.67T-0.81M-0.7{4T}^{2}-1.69M^{2}+0.27T^{3}-0.001M^{3}+0.5{9T}^{4}+1.88M^{4}$$

where y_1_ is the front deviation of the weld, y_2_ is the back deviation of the weld, y_3_ is the average deviation of the weld, T is the spatial position of the welding torch, and M is the mass transfer position.

According to the established regression models, the sum of squares of the total deviation, the sum of squares of the residual and the regression sum of squares of the front deviation, back deviation and average deviation of the weld were calculated, and the significance of each regression coefficient in the model was investigated. Using the analysis of variance (ANOVA) in the Design-Expert software, the significance of the regression coefficient was analyzed to validate the reliability of the model and the regression coefficient. According to the F-test, when “Prob > F” is less than 0.05, the regression coefficient is significant; when “Prob > F” is greater than 0.10, the regression coefficient is not significant; and if the value is between 0.05–0.1, the significance is moderate.

**Figure 6:**
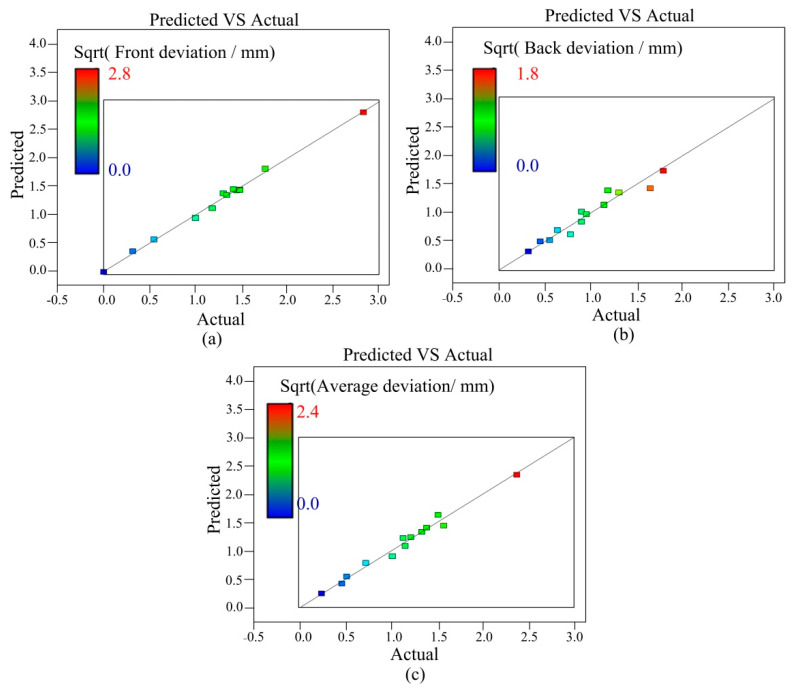
Predicted and actual values: (**a**) front deviation, (**b**) back deviation, (**c**) average deviation.

The F value in Table 5 is 123.49, indicating that the model of the front deviation is significant. The quadratic term (T_2_) of the spatial position of the welding torch, the quadratic term (M_2_) of the mass transfer position, the quartic term (T_4_) of the spatial position of the welding torch and the cubic term (M_3_) of the mass transfer position are moderately significant. The quadratic term (T_3_) of the spatial position of the welding torch and the quadratic term (M_4_) of the mass transfer position are not significant. In Table 6, F = 15.89, suggesting that the model of the back deviation is also significant. In Table 7, F = 27.99, suggesting that the average deviation model is significant. Figure 6 shows the relationship between the predicted and real values of the front deviation, back deviation, and average deviation of the weld seam. All models are significant, and the residual of each prediction response is minimal and follows the diagonal.

### 3.3. Influence of the Two Factors on the Weld Deviation during Variable Position Welding

**Figure 7:**
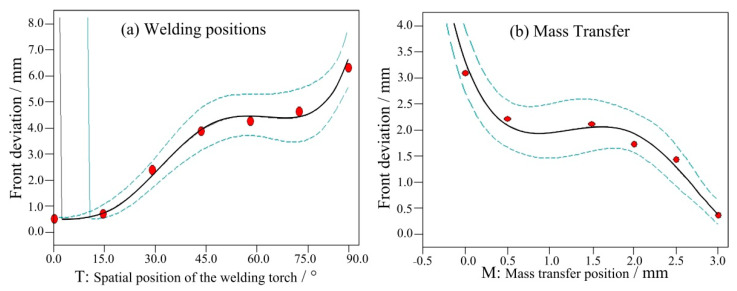
The influence of two factors on the front deviation, (**a**) welding positions and (**b**) mass transfer.

**Figure 8:**
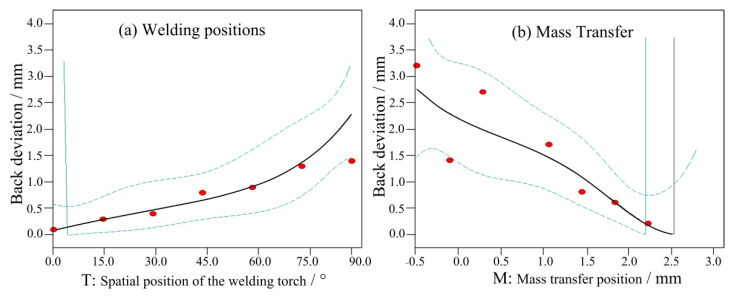
The influence of two factors on the back deviation, (**a**) welding positions and (**b**) mass transfer.

**Figure 9:**
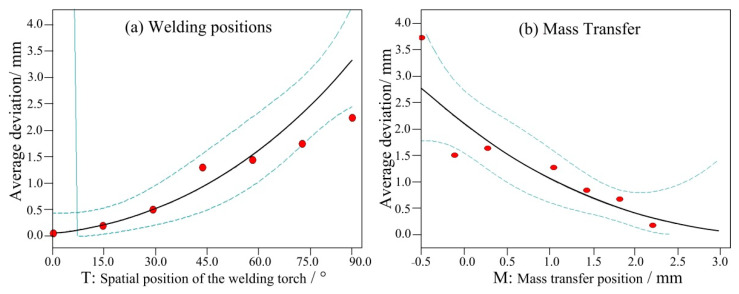
The influence of two factors on the average deviation, (**a**) welding positions and (**b**) mass transfer.

Figure 7 shows the influence of the above two factors on the front deviation of the weld seam. When the mass transfer position was at the centre, the front deviation increased with the welding angle, as shown in Figure 7a. From 0° to 45°, the front deviation increased linearly. Between 45° and 75°, the increase was minimal. From 75° to 90°, the deviation reached its maximum. When the angle was fixed at 90°, the front deviation positively and proportionally changed with the mass transfer position. When the mass transfer position was 3 mm from the central position, the front deviation decreased to less than 0.5 mm. Figure 8 and Figure 9 show the back deviation of the weld seam and the average deviation. When the mass transfer position was fixed, the deviation increased with the welding angle. When the welding angle was fixed, the deviation decreased as the mass transfer position increased. The predicted trajectory was highly consistent with the test results, which proves the significance of the mathematical model for variable position welding.

Based on the actual and predicted results, the deviation and symmetry were largely influenced by the spatial position of the welding torch and the mass transfer position, and the front deviation, back deviation and average deviation were all positively proportional to the welding angle and negatively proportional to the distance between the mass transfer position and the centre position, which is mainly related to the liquid metal flow in the keyhole weld pool. As the angle of the welding torch increased, the angle between the direction of gravity and the welding trajectory increased, and the influence of gravity increased, thereby increasing the weld seam deviation. The mass transfer position can be changed to offset the asymmetric flow caused by gravity by allowing the filler metal to flow to the solidification side of the weld pool through the upper wall, thereby reducing the weld deviation.

**Figure 10:**
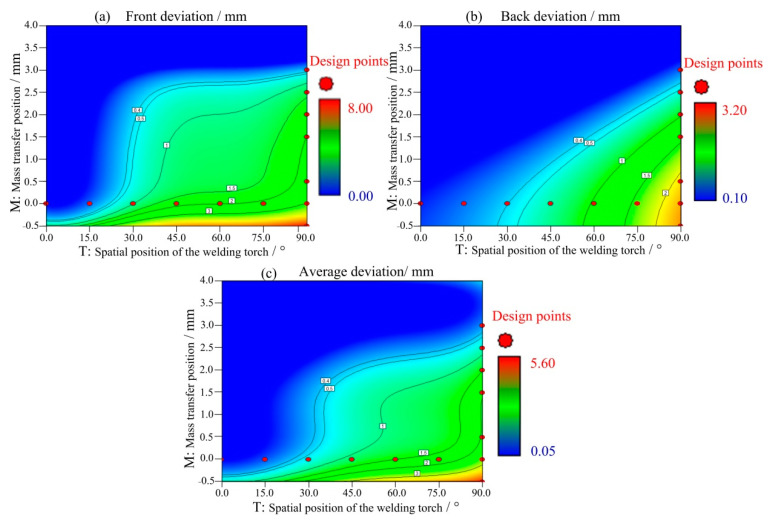
Impact of the interaction between metal transfer and the welding angle on the deviation (**a**) Front deviation, (**b**) Back deviation, (**c**) Average deviation.

Figure 10 presents the contour diagram from Design-Expert, which shows the influence of the mass transfer position and the spatial position of the welding torch on the weld deviation. When the welding position is continuously varied, the mass transfer position needs to be changed to reduce the influence of gravity on the weld formation. Asymmetry can weaken the mechanical properties of welds, and thus, when the welding position is continuously varied, it is of great significance to ensure weld symmetry. However, because there are many uncertain factors in the welding process, this study chose 0.5 mm as the acceptable deviation threshold. Figure 10a shows the contour diagram of the front deviation. When the welding angle was less than 30°, central mass transfer had little impact on the front deviation; after 30°, there was a jump in the front deviation, indicating that maintaining central mass transfer can seriously affect the weld symmetry. According to the mathematical model, to keep the welded joints symmetric (deviation less than 0.5 mm) as the welding position is varied, it is reasonable to maintain central mass transfer when the angle is less than 30°. When the angle exceeds 30°, the mass transfer position should be raised to 2.5 mm to ensure a good weld quality. There was an approximately linear relationship between the back deviation and the mass transfer position, and within 30°, the influence of central mass transfer on the back deviation was minimal. However, the weld symmetry can be negatively affected if central mass transfer is used between 30° and 90°. The average deviation results reveal a trend similar to that of the front deviation of the weld seam.

It is well known that welding is a dynamically stable process, in which external factors have a large influence on the stability and quality of the weld pool. Thus, reducing uncertain influential factors as much as possible throughout the welding process is necessary. According to the optimum results obtained in the experiment and the mathematical models, when the welding position is continuously varied from 0–90°, the mass transfer position should remain unchanged, between 0° and 30° to reduce the influence on the weld pool; from 30° to 90°, the mass transfer position should be adjusted to be 2.5 mm higher than the central mass transfer position to reduce the influence on the weld pool. This method can not only reduce the influence on the weld pool but also ensure that the deviation is within 0.5 mm, which ensures a good weld quality.

## 4. Experimental Results

### 4.1. Macro Morphology of the Weld Seam

Because the direction of gravity does not change, reducing the impact of gravity on the weld pool as the welding position is varied is the key to weld formation. Thus, this study proposed the idea of an asymmetric heat source to address asymmetric flow in variable position welding without changing the parameters. In this section, the entire welding process was optimized based on the optimization strategy proposed in the above section, that is, by using an asymmetric heat source and asymmetric mass transfer to reduce the asymmetric weld pool flow caused by gravity during variable position welding. The results provide technical support for the actual application of variable position welding.

The specific steps of applying the asymmetric heat source are as follows: The heat source is at an angle (15°) relative to the thickness direction during variable position welding, which is the angle between the axis of the welding torch and the welding direction, and the welding torch pushes the liquid metal and moves it forward. The asymmetric heat source, without changing the welding parameters, can ensure keyholing on the molten side, reduce the curvature radius of the hole on the solidification side, and enhance the surface tension between the radial force of the plasma arc and the liquid metal, which reduces the influence of gravity on the weld pool flow and the direct influence of the axial force of the arc. In addition, the large thermal gradient on the solidification side ensures that the metal easily solidifies and forms the welds, providing an optimal heat source for variable position welding. The asymmetric heat source enables the welding position to be varied with a reliable and stable thermal environment, yet the asymmetric flow caused by the change in the direction of gravity is not changed.

The specific steps of asymmetric mass transfer are as follows: Asymmetric weld pool flow can result in an uneven thermal distribution, thereby affecting the mechanical properties of welded joints. During variable position welding, liquid metal with a high temperature flows to the lower side of the weld pool, causing a difference in the thermal cycle curve between the two sides of the weld, and the highest temperature of the thermal cycle on the lower side is higher than that on the upper side of the weld pool. Therefore, during variable position welding, asymmetric mass transfer is introduced to solve this problem. The wires should be higher than the stagnation point on the molten side of the weld pool so that most molten metal flows from the upper wall of the weld pool to the solidification side, thereby reducing the asymmetric flow due to the influence of gravity and reducing the difference in the thermal cycle on both sides of the weld. The following results were obtained. According to the optimum results obtained in the experiment and the mathematical model, as the welding position continuously varies from 0–90°, the mass transfer position should remain unchanged between 0° and 30° to reduce the influences on the weld pool; from 30° to 90°, the mass transfer position should be adjusted to be 2.5 mm higher than the central mass transfer position to reduce the influence on the weld pool. This method can not only reduce the influence on the weld pool but also ensure that the deviation is within 0.5 mm, which ensures good weld quality.

**Figure 11:**
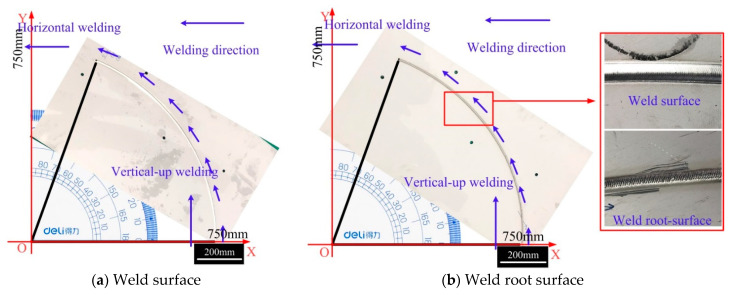
Weld appearance from vertical-up to horizontal welding. (**a**) Weld surface, (**b**) Weld root surface.

**Figure 12:**
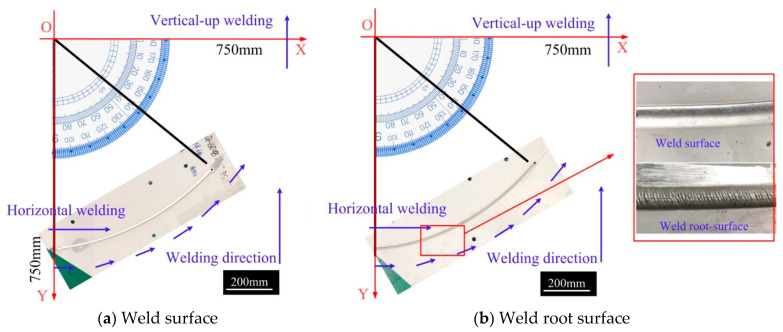
Weld appearance from horizontal to vertical-up welding. (**a**) Weld surface, (**b**) Weld root surface.

Figure 11 shows the results of variable position welding for a 5 mm plate from a vertical-up to a horizontal position using the asymmetric heat source and the optimized mass transfer position. The welding started with the stable vertical-up welding and ended when the angle between the direction of gravity and the welding direction was 70°. During the entire welding process, the weld appearance was good, without obvious defects. Figure 12 shows the results of variable position welding for 5 mm and 3 mm plates from a vertical-up to a horizontal position using the asymmetric heat source and asymmetric mass transfer. As Figure 12 shows, in the initial stage, the liquid bridge was stably established on the solidification side of the weld pool, and the weld appearance was good.

### 4.2. Morphology and Defects of Welded Joints

**Figure 13:**
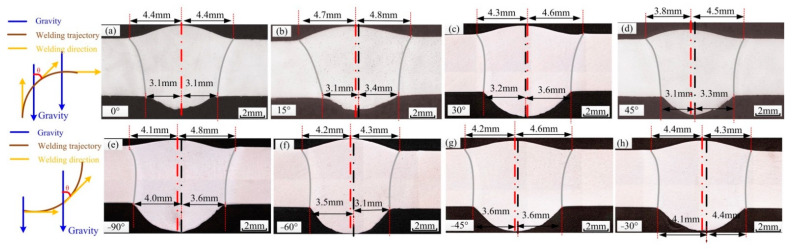
Cross section of the welded joint after optimization, red line is the center line of weld surface, black line is the center line of weld root-surface. (**a**) 15°, (**b**) 30°, (**c**) 45°, (**d**) 60°, (**e**) −75°, (**f**) −60°, (**g**) −45°, (**h**) −30°.

Figure 13 shows the welded joints at different angles obtained using the optimized variable position welding strategy. Figure 13a,b shows the transition stage from vertical–up welding to horizontal welding, during which the welded joints were symmetric. When the angle was approximately 60°, there was a slight front deviation. In the transition from vertical-up welding to horizontal welding, the maximum front deviation was 0.7 mm, and the maximum back deviation was 0.4 mm. In the transition from horizontal welding to vertical-up welding, the keyhole weld pool was established stably at −90°, with a stable liquid bridge on the solidification side and good weld formation. In addition, the maximum front deviation was 0.7 mm, and the maximum back deviation was 0.4 mm.

**Figure 14:**
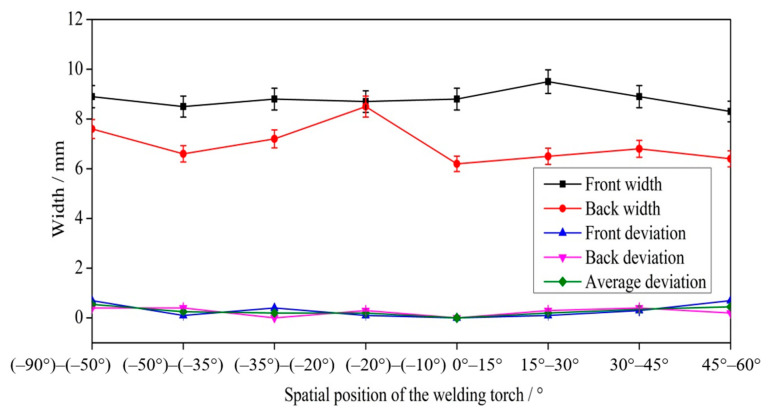
The size and deviation of welded joints after optimization.

Figure 14 shows the size and deviation of welded joints after optimization. Because the welding parameters were the same, the size of the welded joints remained stable as the welding position varied. However, due to the optimized asymmetric heat source, the curvature of the solidification side was smaller than that of the molten side and smaller than that of the solidification side when using a symmetric heat source. Thus, the size of the welded joints after optimization was smaller than that when using a symmetric heat source. The asymmetric heat source reduced the curvature of the solidification side of the weld pool and the equivalent arc length of the solidification side; thus, the attenuation of the arc energy density in the thickness direction was minimal, thereby increasing the curvature at the outlet of the solidification side. The width of the front excess weld metal height after optimization was between 8.3 mm and 9.5 mm, and the width of the back excess weld metal height was between 6.4 mm and 8.5 mm. At −20°, the widths of the front and back excess weld metal heights were similar, possibly because the small keyhole weld pool was disturbed when the mass transfer position was adjusted. Before optimization, the deviation of the weld seam was large, especially when the angle between the direction of gravity and the welding direction was large. The overall deviation after optimization decreased, the deviation when the welding angle was 0° remained within 0.7 mm, and the weld appearance was good, without any obvious defects.

### 4.3. Mechanical Properties of Welded Joints

**Figure 15:**
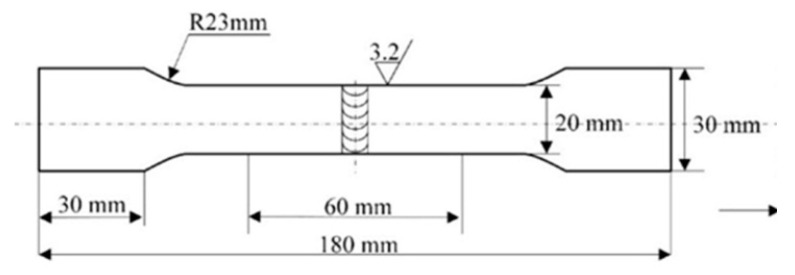
Schematic diagram for geometrical dimensions of test samples.

**Figure 16:**
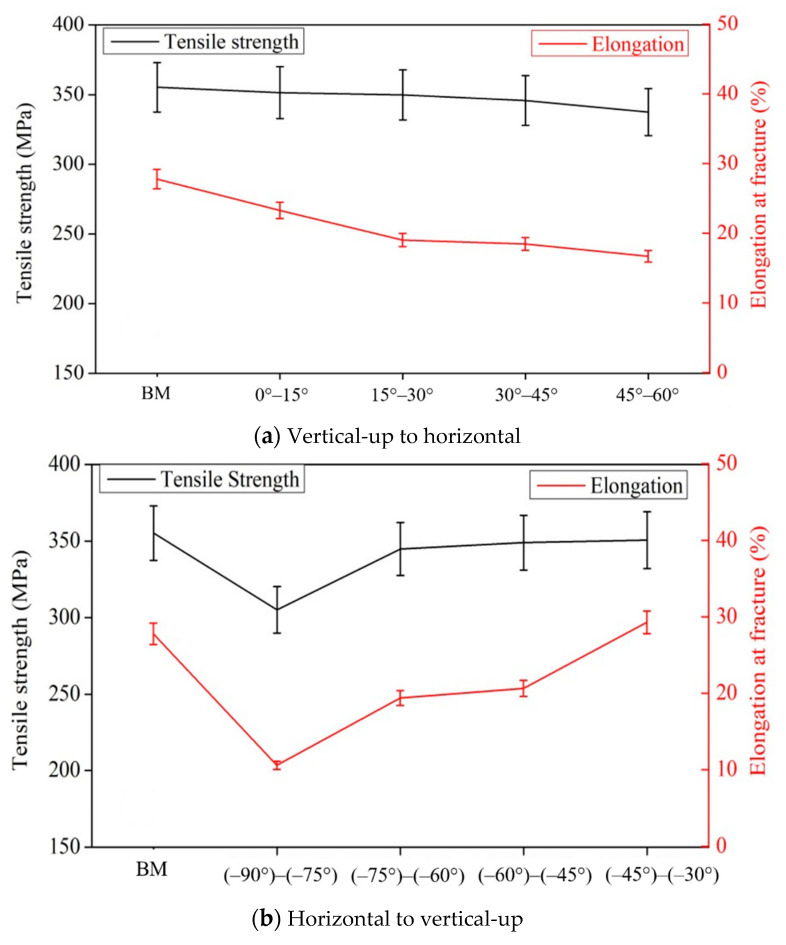
Mechanical properties after optimization. (**a**) Vertical–up to horizontal welding, (**b**) Horizontal to vertical-up welding.

Tensile tests were carried out at room temperature using a universal testing machine INSTRON-5569 under standard NoGBT228–2002 of P.R. China [2]. The size of the dog-bone specimen used to measure the tensile strength is shown in Figure 15. The results are taken as the average value of three samples. Figure 16 shows the mechanical properties of the welded joints after optimization. During the transition from vertical–up welding to horizontal welding, the mechanical properties of the weld remained at over 90% of those of the base material. During the transition from horizontal welding to vertical-up welding, the weld seam with the smallest tensile strength was horizontal and approximately 85.9% of the base material. Before optimization, during the variable position welding process, only the weld seam between −20° and −30° was of good quality; that is, the welding quality met the standard only when the welding angle was not largely different from that of vertical-up welding, and the spatial range of the welding torch that met the standard welding quality was small. After optimization, the spatial range of the welding torch that met the standard welding quality increased to 0°.

When the welding position is continuously varied, the keyhole weld pool cannot bear the impact caused by the changes in the direction of gravity, which makes the weld pool unstable and even causes it to collapse and cut. Because the direction of gravity does not change, reducing the impact of gravity on the weld pool is the key to weld formation during variable position welding. An angle between the direction of gravity and the welding direction was applied to push the liquid metal. This method keeps the keyhole ability of the molten pool without changing the welding parameters [25]. It also can reduce the radius of the keyhole on the solidification side, enhance the arc radial force and surface tension to weaken the influence of gravity on the molten pool flow. In addition, the larger thermal gradient on the solidification side makes the metal easy to solidify and form a weld, which provides an ideal heat source for variable position welding [25,26]. Porosity is easily formed in the solidification process of non-vertical up welding, this kind of pores are mainly hydrogen pores, the key reason for the formation of hydrogen pores is the difference in the solubility of hydrogen in the solid and liquid metal [13]. Previous studies have proposed the idea of an asymmetric heat source for solving asymmetric flow and reduced porosity, which provides a theoretical basis for improving variable position welding without changing the welding parameters [2,13,17]. The weld formation during variable position welding is the premise of practical applications, and elimination of the welding defects caused by asymmetric flow is the goal. Although an asymmetric heat source can improve the weld formation, asymmetric flow of the weld pool still exists in actual variable position welding applications. The asymmetric weld pool flow can result in an uneven thermal distribution, thereby affecting the mechanical properties of the welded joints. The grain size of the weld after heat treatment is inversely proportional to the pool cooling rate ($\Delta T_{f}$ undercooling) When other conditions are the same. Since the initial temperature of the both sides of the weld is consistent with the final temperature, the grain size of the both sides of the weld is inversely proportional to the maximum temperature of the thermal cycle, that is, the higher the maximum temperature, the smaller the grain size, thus affecting the performance of the welded joint [2,17,26]. During variable position welding, liquid metal with a high temperature flows to the lower side of the weld pool, causing a difference in the thermal cycle curve between the two sides of the weld, and the highest temperature of the thermal cycle on the lower side is higher than that on the upper side of the weld pool. The asymmetric thermal cycle is due to the influence of gravity [17]. Thus, reducing the asymmetric flow of the weld caused by gravity is the key to improving the mechanical properties of the weld seam during variable position welding. Asymmetric mass transfer was introduced in this study to ensure that more metal flowed from the upper side to the solidification side, thereby optimizing the asymmetry.

**Figure 17:**
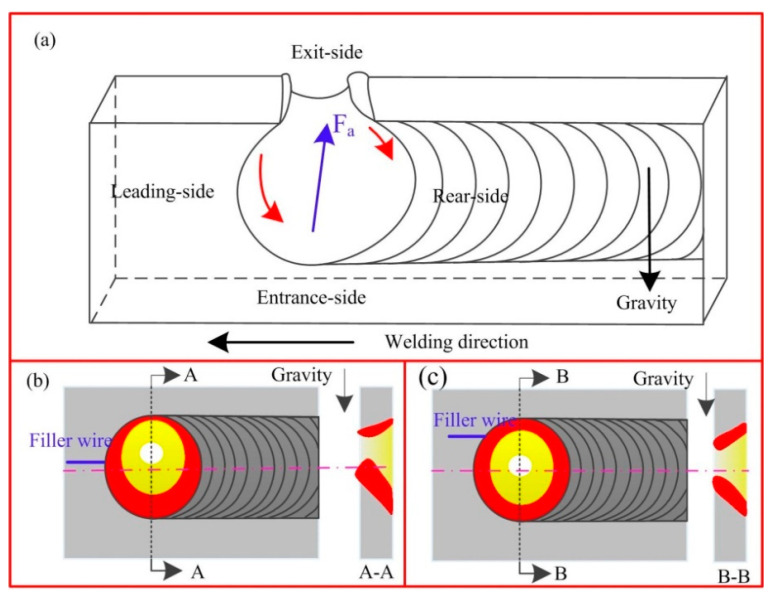
Schematic of molten metal in a weld pool with a keyhole. (**a**) 3D diagram, (**b**) traditional mass transfer, (**c**) asymmetric mass transfer.

As shown in Figure 17, if the traditional mass transfer position is used during horizontal welding, as the heat source moves, most liquid metal flows from the wall of the hole to the lower side, and most of the heat is transferred. During solidification, the side with more molten metal has a higher temperature, which was validated by both experimental and theoretical results. Therefore, the highest temperature on the lower side of the horizontal welded joints under traditional mass transfer was higher than that of the upper side. When using asymmetric mass transfer, especially when the mass transfer position is higher than the flow stagnation point on the molten side (the flow stagnation point of the weld pool was determined via numerical simulation, direct observation of the weld pool flow, and indirect descriptions of the weld pool flow trajectory in Section 3), molten wires flow from the upper wall of the weld pool to the solidification side, resisting the asymmetric flow caused by gravity (Figure 17c). Thus, the thermal cycle curves on both sides of nonvertical-up welded joints with asymmetric mass transfer were greatly improved.

## 5. Conclusions

In this study, a continuously variable welding position experiment was designed using the RSM, and the relationships between the weld morphology and both the mass transfer position and the position of the welding torch were obtained. By comparing the morphology and mechanical properties of welded joints before and after optimization, the idea of eliminating asymmetric welded joints by using an asymmetric heat source and asymmetric mass transfer was validated. The conclusions are as follows:

(1) The relationships between the weld morphology and both the mass transfer position and the spatial position of the welding torch were established, and the accuracy and reliability of the models were analyzed via ANOVA. The fitted curves of the front, back, and average deviations of the weld seam were all similar to the actual curves.

(2) After considering weld stability, the optimal solution was obtained based on the mathematical models. To reduce the influence on the weld pool, the mass transfer position was adjusted to be 2.5 mm higher than the central mass transfer position, and the deviation was smaller than 0.5 mm, which ensured a good weld quality.

(3) A 5 mm plate was welded with a continuously varied position after optimization was conducted, and the weld appearance was good. The mechanical properties of the weld reached at least 85% those of the base material, thus meeting the requirements of Al-alloy welding.

(4) The process control of the keyhole weld pool from fixed environmental variables to dynamic environmental variables was realized, which enabled the Al-alloy keyhole weld pool to resist the disturbance caused by the changes in the welding environment during variable position welding and ensured a good weld quality.

## 6. Outlook

This paper used 5A06 as the base metal, the applicability of variable position welding for different materials should be studied to further expand the application of VPPA Al alloys keyhole welding. In addition, the research mainly focused on the stability of the keyhole molten pool caused by the change of welding direction in a plane, but the state of the molten pool and weld quality under the continuous variable force in space has not been verified.

## Figures and Tables

**Table 1:** Chemical composition of 5A06 and ER5183 (wt.%) [22,23].

Materials	Mg%	Mn%	Cr%	Fe%	Si%	Zn%	Cu%	Al%
5A06 plate	5.80~6.80	0.50~0.80	--	<0.40	≤0.40	≤0.20	≤0.10	Bal.
ER5183	4.30~5.00	0.50~1.00	≤0.1	≤0.40	≤0.40	≤0.25	≤0.10	Bal.

**Table 2:** Welding parameters.

Parameter		Value/Unit
Welding current	DCEN	130 A
	DCEP	155 A
Welding mode		Keyhole welding
Welding angles		Continuously Varying Positions
Travel speed		0.15 m/min
Wire feed speed		0.8 m/min
Wire diameter		1.2 mm
Shielding gas flow rate		Pure Ar with 15 L/min
Plasma gas flow rate		Pure Ar with 3.0 L/min

**Table 3:** Coded values and factor levels.

Factor	Unit	Value				
		−1.414	−1	0	1	1.414
Spatial welding position	°	90	75	45	30	0
Mass transfer position	mm	−0.5	0	1.5	2.5	3.5

**Table 4:** Central composite design matrix and deviation.

Std Group	Rum No.	Factor 1	Factor 2	Response 1	Response 2	Response 3
		Spatial Welding Position	Mass Transfer Position	Front Deviation	Back Deviation	Average Deviation
		°	mm	mm	mm	mm
2	#1	0.00	0.00	0.0	0.1	0.05
3	#2	15.00	0.00	0.1	0.3	0.20
10	#3	30.00	0.00	1.0	0.0	0.50
6	#4	45.00	0.00	1.8	0.8	1.30
13	#5	60.00	0.00	2.0	0.9	1.45
1	#6	75.00	0.00	2.2	1.3	1.75
7	#7	90.00	0.00	3.1	1.4	2.25
12	#8	90.00	−0.50	8.0	3.1	5.60
9	#9	90.00	0.50	2.2	2.7	2.45
5	#10	90.00	1.50	2.1	1.7	1.90
4	#11	90.00	2.00	1.7	0.8	1.25
11	#12	90.00	2.50	1.4	0.6	1.00
8	#13	90.00	3.00	0.3	0.2	0.25

**Table 5:** ANOVA results for the surface deviation model.

Source	Sum of Squares	*D_f_*	Mean Square	F Value	*p* Value Prob > F	
Model	5.91	8	0.74	123.49	0.0002	Significant
T-spatial welding position	0.24	1	0.24	39.49	0.0033	--
M-mass transfer position	3.55 × 10^−3^	1	3.55 × 10^−3^	0.59	0.4841	--
T2	0.12	1	0.12	20.52	0.0106	--
M2	0.13	1	0.13	20.95	0.0102	--
T3	9.13 × 10^−3^	1	9.13 × 10^−3^	1.53	0.2843	--
M3	0.03	1	0.03	5.40	0.0807	--
T4	0.07	1	0.07	12.02	0.0257	--
M4	7.77 × 10^−3^	1	7.77 × 10^−3^	1.13	0.3179	--
Residual	0.02	4	5.98 × 10^−3^	--	--	--
Cor total	5.93	12	--	--	--	--

**Table 6:** ANOVA results for the root surface deviation model.

Source	Sum of Squares	D_f_	Mean Square	F Value	*p* Value Prob > F	
Model	2.09	8	0.52	15.89	0.0007	Significant
*T*-spatial welding position	1.44	1	1.44	43.79	0.0002	--
*M*-mass transfer position	0.72	1	0.72	22.06	0.0015	--
T2	0.03	1	0.03	0.92	0.3647	--
M2	0.05	1	0.05	1.63	0.2370	--
Residual	0.26	8	0.03	--	--	--
Cor total	2.35	12	--	--	--	--

**Table 7:** ANOVA results for the average deviation model.

Source	Sum of Squares	*D_f_*	Mean Square	F Value	*p* Value Prob > F	
Model	3.77	8	0.47	27.99	0.0030	Significant
*T*-spatial welding position	0.19	1	0.19	11.55	0.273	--
M-mass transfer position	0.061	1	0.06	3.60	0.1305	--
T2	0.031	1	0.03	1.86	0.0244	--
M2	0.12	1	0.12	7.68	0.0503	--
T3	1.59 × 10^−4^	1	1.59 × 10^−4^	1.53	0.9272	--
M3	4.41 × 10^−7^	1	4.41 × 10^−7^	5.40	0.0996	--
T4	0.02	1	0.02	1.44	0.0257	--
M4	0.03	1	0.03	1.83	0.0248	--
Residual	0.07	4	0.02	--	--	--
Cor total	3.84	12	--	--	--	--

## Data Availability

Not applicable.
